# Supplementary material for: Tropical peat composition may provide a negative feedback on fire occurrence and severity
Source: Nat Commun. 2024 Aug 27;15:7363. doi: 10.1038/s41467-024-50916-7 (PMC11349947; doi:10.1038/s41467-024-50916-7)
Supplement: Supplementary file 1 — Supplementary Information [file 41467_2024_50916_MOESM1_ESM.pdf]

Supplementary Information

Tropical peat composition may provide a negative feedback on fire occurrence and severity

Crawford et al.

Table S1: Locations, climatic regions, and sampling depths for the peat coring sites.

Site number	Site name / location	Latitude	Longitude	Climatic region	Aerobic layer sample depths (cm)	Anaerobic layer sample depths (cm)
1	Western Svalbard, Norway	78.933 ° N	11.483 °E	Arctic	–	19
2	Northern Alaska, USA	69.541 ° N	148.602 °W	Arctic	5	50
3	Northern Alaska, USA	69.536 ° N	148.574 °W	Arctic	5	50
4	Northern Alaska, USA	69.421 ° N	148.691 °W	Arctic	5	50
5	Northern Alaska, USA	69.323 ° N	148.721 °W	Arctic	5	50
6	Hanhijanka, Finland	69.167 ° N	27.150 °E	Boreal	12	51
7	Northern Alaska, USA	68.965 ° N	148.084 °W	Arctic	5	50
8	Northern Alaska, USA	68.814 ° N	148.841 °W	Arctic	5	50
9	Toolik, Alaska, USA	68.620 ° N	149.600 °W	Arctic	5, 5, 5	50, 50, 50
10	Northern Alaska, USA	68.619 ° N	149.325 °W	Arctic	5	50
11	Northern Alaska, USA	68.458 ° N	149.346 °W	Arctic	5	50
12	Luovuoma, Finland	68.400 ° N	23.433 °E	Boreal	12	21, 51
13	Crater Pool, Sweden	68.319 ° N	19.858 °E	Boreal	3, 5, 5, 11	19, 20, 28
14	Instrument Bog, Sweden	68.183 ° N	19.750 °E	Boreal	2	26
15	Railway Bog, Sweden	68.083 ° N	19.817 °E	Boreal	2, 5	23, 40, 41
16	Marooned, Sweden	67.957 ° N	19.987 °E	Boreal	5	36
17	Electric Bog, Sweden	67.850 ° N	19.367 °E	Boreal	2	22
18	Indico, Russia	67.275 ° N	49.880 °E	Arctic	9	35
19	Hautasuo Tupakkisuo, Finland	65.650 ° N	27.050 °E	Boreal	–	21, 23, 51, 51
20	Saarisuo, Finland	65.550 ° N	27.533 °E	Boreal	–	21, 51
21	Nadym, Russia	65.333 ° N	72.917 °E	Boreal	6	24
22	Haukkasuo, Finland	60.817 ° N	26.900 °E	Boreal	–	21, 51
23	Underhoull, Shetland, Scotland, UK	60.719 ° N	0.947 °W	Temperate	2	45
24	Fagelmossen, Sweden	59.533 ° N	12.183 °E	Temperate	5	50, 153
25	North Uist, Scotland, UK	57.567 ° N	7.300 °W	Temperate	–	33
26	Shestakovo, Russia	55.883 ° N	87.833 °E	Temperate	6	31
27	Slieveanorra, Northern Ireland, UK	55.085 ° N	6.192 °W	Temperate	10	64
28	Walton Moss, England, UK	54.993 ° N	2.764 °W	Temperate	5	50, 373

29	Fallahogy Bog, Northern Ireland, UK	54.750 ° N	6.600 °W	Temperate	3, 5	50, 423
30	Dosenmoor, Germany	54.138 ° N	10.025 °E	Temperate	5	50, 468
31	All Saints Bog, Ireland	53.150 ° N	7.983 °W	Temperate	15	–
32	Migneint, Wales, UK	52.970 ° N	3.840 °W	Temperate	6	26
33	Lac-à-la-Truite, Blanc Sablon, Quebec, Canada	51.483 ° N	57.183 °W	Boreal	5, 5	50, 50
34	Burnt Village Bog, Newfoundland, Canada	51.130 ° N	55.930 °W	Boreal	5	51
35	Manacrin Moor, England, UK	50.533 ° N	4.617 °W	Temperate	–	58
36	Plaine Bog, Quebec, Canada	50.274 ° N	63.541 °W	Boreal	–	26
37	Morts, Havre-St-Pierre, Quebec, Canada	50.267 ° N	63.667 °W	Boreal	5, 5	50, 50
38	Lebel, Baie Comeau, Quebec, Canada	49.100 ° N	68.233 °W	Boreal	5, 5	50, 50
39	Petite Bog, Nova Scotia, Canada	45.151 ° N	63.939 °W	Temperate	5	51
40	Sidney Bog, Maine, USA	44.388 ° N	69.788 °W	Temperate	5	51
41	Piyashiri, Honshu, Japan	36.017 ° N	139.717 °E	Temperate	6	51, 121
42	Oropel Swamp, Panama	9.380° N	82.370 °W	Tropical	5	50
43	Sebangau Swamp Forest, Kalimantan, Indonesia	2.316° S	113.887 °E	Tropical	2	29
44	Sebangau Swamp Forest, Kalimantan, Indonesia	2.321° S	113.884 °E	Tropical	2, 6, 6	29, 51, 51
45	Sebangau Swamp Forest, Kalimantan, Indonesia	2.323° S	113.904 °E	Tropical	2	29
46	Quistococha, Peru	3.840° S	73.319 °W	Tropical	0	50, 200, 369, 390
47	Aucayacu, Peru	3.937° S	74.385 °W	Tropical	4, 5	50, 57
48	San Jorge, Peru	4.050° S	73.183 °W	Tropical	–	45, 80, 149, 241
49	Buena Vista, Peru	4.233° S	73.200 °W	Tropical	–	30, 314
50	Miraflores, Peru	4.410° S	74.063 °W	Tropical	0, 0	32, 32
51	Nueva York, Peru	4.417° S	74.280 °W	Tropical	0, 0	32, 32
52	Ollanta, Peru	4.450° S	74.860 °W	Tropical	0, 0, 0, 0	32, 32, 32, 32
53	San Roque, Peru	4.544° S	74.626 °W	Tropical	0	–
54	Karukinka, Chile	53.860 ° S	69.576 °W	Temperate	6	51
55	Tierra Australis, Argentina	54.616 ° S	67.771 °W	Temperate	6	51
